# Beyond Suppression: Peripheral T Cell Responses to Vaccination in Inflammatory Bowel Disease Patients Undergoing Anti-Tumor-Necrosis-Factor Therapy

**DOI:** 10.3390/vaccines12111280

**Published:** 2024-11-14

**Authors:** Martin Qui, Ennaliza Salazar

**Affiliations:** 1 Duke-NUS Medical School, Singapore 169857, Singapore; 2 Department of Gastroenterology and Hepatology, Singapore General Hospital, Singapore 169608, Singapore

**Keywords:** inflammatory bowel disease, immunomodulation, T cell, adaptive immunity, TNF

## Abstract

Alimentary tract inflammation in inflammatory bowel disease (IBD) is treated by systemically administered drugs that alter fundamental host immune responses. Biologics that target tumor necrosis factor (TNF) are first-line biologics in IBD, used widely for their effectiveness, steroid-sparing quality, and lower cost. While they enable a significant proportion of patients to achieve clinical remission, they carry an increased risk of infection and poor serological responses to vaccination. Conversely, our understanding of adaptive T cell responses in anti-TNF-treated IBD patients remains limited. The introduction of COVID-19 vaccines has prompted research that both challenges and refines our view on immunomodulatory therapy and its potential implications for immunity and protection. Here, we review these emergent findings, evaluate how they shape our understanding of vaccine-induced T cell responses in the context of anti-TNF therapy in IBD, and provide a perspective highlighting the need for a holistic evaluation of both cellular and humoral immunity in this population.

## 1. Introduction

Crohn’s disease (CD) and ulcerative colitis (UC) comprise the inflammatory bowel diseases, characterized by the progressive immunopathologic destruction of the alimentary tract. IBD manifests in affected individuals as symptoms spanning from distressing abdominal pain and diarrhea to serious complications resulting in bowel obstruction, perforation, or even malignancy. In 2019, global estimates for people living with IBD were at 4.9 million, an increase of almost 50% since 1990 [1], and numbers are only expected to rise with the rapid changes in newly industrialized countries [2].

The treatment of IBD relies on the targeting of dysregulated pro-inflammatory pathways that have been identified in recent decades, which are reviewed in detail elsewhere [3]. Recent evidence, particularly in CD, favors rapid treatment escalation from corticosteroid induction to advanced biologics in order to achieve clinical disease remission and mucosal healing [4]. While the widespread use of anti-TNF-based maintenance therapies are practicable and revolutionary in IBD treatment [5], their potential for immunosuppression has raised concerns. The risk of latent tuberculosis [6] or hepatitis B virus reactivation [7] is routinely screened for prior to the initiation of anti-TNF- and anti-p40-based therapies. Moreover, an increased risk of opportunistic infections with Candida and herpesvirus infections has been reported [8], with guidelines even advising against inoculation with live-attenuated vaccines due to the risk of pathologic infection [9].

Vaccination offers an important strategy to protect IBD patients undergoing immunomodulatory treatment against infectious disease, with the caveat that vaccination fundamentally depends on immunological pathways that overlap with those necessary for responding to infections. The immunogenicity of several vaccines, along with their subsequent protective efficacy in anti-TNF-treated IBD patients, has therefore been put into question. Our understanding of the effect of such therapies is mainly shaped by studies that quantitatively assessed seroconversion following vaccination in anti-TNF-treated patients, largely pointing towards their suppressive nature. Indeed, most vaccines are designed to induce protective humoral responses [10], and therefore it seems sensible to presume that the lower antibody titers achieved by vaccination in such patients indicate that they are defective, unlike in healthy, untreated individuals. Lower rates of seroconversion in response to various vaccines have been observed specifically in patients on anti-TNF therapy [11], suggesting that T-dependent responses are hindered more than T-independent B cell responses in anti-TNF therapy [12].

However, such findings do not preclude the development of productive T cell responses that may aid in protection against infectious disease. In contrast to vaccine-induced serological responses, this area has yet to be scrutinized as closely, particularly in patients with IBD. The COVID-19 pandemic has further highlighted the importance of T cell responses in controlling SARS-CoV-2 infection in the midst of antibody escape and the rapid waning of responses, prompting extensive studies on their induction and properties post COVID-19 vaccination [13]. Importantly, new findings involving vaccine-induced T cell responses detected in patients with IBD have surfaced, prompting a re-evaluation of our view on such therapies.

With this in mind, we will review the recent literature shaping our current understanding of the vaccine-induced immune responses in patients with IBD undergoing immune-modifying therapies. First, we will highlight the roles and functions of T cells in host protection against infectious disease. Then, we will explore the recent evidence from studies focusing on vaccine-induced T cell responses in anti-TNF-treated IBD patients that challenge the prevailing view that such therapies exert general suppressive effects on their development of functional immune responses and contrast these with other widely used treatments. Finally, we will summarize this review and discuss the unanswered questions/gaps in this field.

## 2. The Immunological Niche of T Cells in Infection and Disease

T cells are integral components of cell-mediated adaptive immunity that exhibit a high level of specialization. Mechanistically, T cells occupy the role of direct intracellular pathogen control, limiting their proliferation within infected host cells and subsequent spread. Their specificity is based upon the recognition of pathogen-derived peptides displayed on major histocompatibility complexes (MHCs) on the surface of both infected cells and antigen-presenting cells (APCs) [14,15], leading to the execution of their effector function. This contrasts with the role of humoral antibodies that neutralize pathogens and their products through direct binding within the extracellular space. Two distinct T cell subpopulations, CD4+ and CD8+, play crucial roles during infections and complement the ability of the innate immune system to curb initial pathogen replication and cooperate with antibodies in preventing the infection of new cells [16].

CD4+ T cells orchestrate adaptive immune responses by producing pro-inflammatory cytokines in response to antigens presented on MHC class II complexes of APCs [17]. Following an antigenic stimulus, these cells commit to one of a wide variety of phenotypes characterized by their secreted products and functions. These include those that specialize in intracellular pathogen clearance (T_H_1), extracellular pathogen responses (T_H_2), barrier maintenance (T_H_17), B cell activation and maturation (T_FH_), or even antigen tolerance (T_REG_) [18]. Many of these states, which are well or recently characterized, continue to undergo definition and refinement [19].

While functional diversity is attributed to CD4+ T cells, CD8+ T cells function predominantly in the cytolytic clearance of cells that display a foreign antigen derived from intracellular pathogens presented on MHC class I complexes. Ultimately, target cell ligation by CD8+ T cell receptors (TCRs) induce the apoptosis of target cells. Since MHC class I is widely expressed on all nucleated cells, an extensive array of cell types may be subject to CD8+ T cell-mediated cytolysis [20]. The widely available vaccines provide the necessary stimuli to induce antigen-specific CD4+ and CD8+ T cell subsets to varying degrees, with the latter more robustly induced by vaccine designs that utilize the host translation machinery in order to feed antigens into the MHC class I processing pathway, such as viral vector, live-attenuated virus, or more recently, messenger RNA (mRNA) vaccines [21].

### 2.1. The Geographic Niches of T Cells

An important caveat in many studies on human immunological responses in the peripheral blood is that the distribution of T cell subpopulations varies vastly within anatomic compartments. Quantities of CD4+ and CD8+ subsets do not correspond proportionally between blood and most peripheral tissues [22], with T cell quantities predominating within the lymphatic system [23]. It is also becoming increasingly recognized that both memory CD8+ and CD4+ T cells are enriched mainly throughout the tissues as resident memory T cells (T_RM_) rather than as circulating peripheral blood mononuclear cells [24,25,26,27]. Importantly, residence in such sites is established independent of the presence of a sustained antigen [28]. Their strategic localization was shown to be important in early host protection and was demonstrated in pre-clinical models of respiratory influenza [29,30,31] or coronaviral [32] infections. Notably, their induction appears to be restricted to antigen priming within the involved tissues themselves and not with systemic immunization [33,34,35]. Several studies that sampled mucosal sites such as the upper and lower respiratory tracts show that intramuscular COVID-19 vaccination does not induce T cells that persist in barrier sites [34,35,36]. These suggest that in the context of assessing immunological responses to COVID-19 vaccines, probing vaccine-induced responses within the peripheral blood may be reasonable and practical.

### 2.2. T Cells in Infection and Disease

In the pre-COVID-19 era, the detection of antigen-specific CD4+ and CD8+ T cell subsets post vaccination was linked to protection against some vaccine-preventable diseases. Bacille Calmette–Guérin (BCG) immunization of infants induces BCG-specific T cells that secrete T_H_1 cytokines, and their detection is associated with a reduced risk of developing tuberculosis [37]. Meanwhile, the rapid airway accumulation of BCG-specific CD8+ T cells was highly correlated with protection against aerosolized Mycobacterium tuberculosis [38]. Detailed analysis of influenza-vaccinated and unvaccinated individuals implicated polyfunctional effector T cell populations as the principal correlate of protection from symptomatic influenza [39].

More recently, the impact of vaccine-induced T cells in the control of SARS-CoV-2 infection was determined, although clear quantitative thresholds of T cell parameters associated with protection are lacking. Importantly, many of these observations were made by linking the evolving virological properties of SARS-CoV-2 that imparted properties of antibody escape [40] with the continued protection that COVID-19 vaccination afforded [41,42]. This was first established by studies in non-human primates, in which CD8+ T cell depletion impaired the ability of adaptive immunity to control experimental SARS-CoV-2 infection in rhesus macaques [43]. In later human studies, breakthrough SARS-CoV-2 infection was demonstrated to activate and increase SARS-CoV-2-specific CD8+ T cells [44], as well as CD4+ T cells [45] that correlated with reductions in upper respiratory tract viral load.

In terms of protection from illness, those who suffered or even succumbed to severe COVID-19 exhibited poor viral control within their upper respiratory tracts, paralleled by a delay in the induction of T cell responses quantified in the peripheral blood by interferon gamma (IFN-γ) ELISpot, and lower detected T cell frequencies. In contrast, controllers with only mild COVID-19 exhibited an earlier and quantitatively robust T cell response [46]. The demonstration of the role of T cell responses in protection was likewise demonstrated in vaccine- and infection-naïve individuals who exhibited cross-protective T cells in their circulation. An enrichment of SARS-CoV-2-specific T cells was discovered in healthcare workers who demonstrated resistance to SARS-CoV-2 infection and remained persistently seronegative despite repeated exposures in high-risk settings [47]. Interestingly, HLA-B*15:01 status was associated with asymptomatic SARS-CoV-2 infection and linked to the presence of a cross-reactive CD8+ T cell that recognizes the SARS-CoV-2 Spike-derived NQKLIANQF/HLA-B*15:01 complex [48].

## 3. Anti-TNF and the Induction of Antigen-Specific T Cell Responses

**Figure 1:**
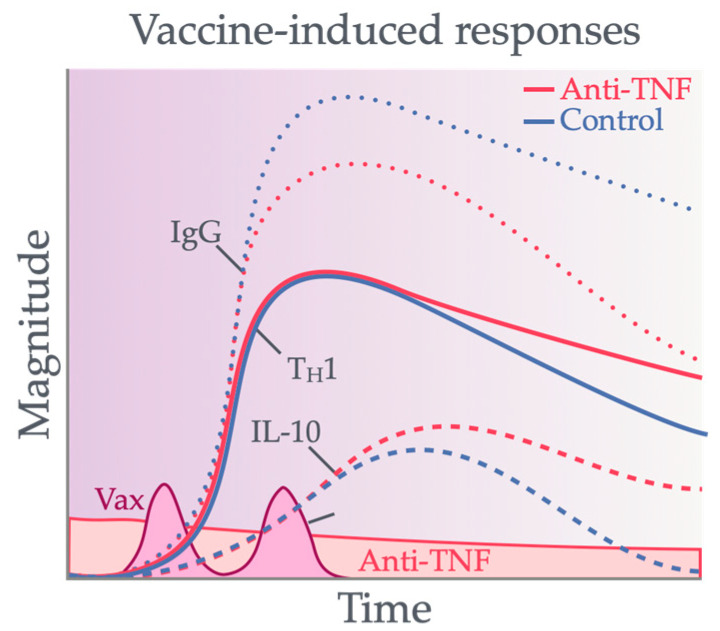
Magnitude of vaccine-induced adaptive immune responses in patients treated with anti-TNF therapy compared to healthy controls. Despite the defective induction of vaccine-induced humoral (IgG) responses, cellular T_H_1/IL-10 responses persist. The line styles represent the different types of vaccine-induced responses measured, as labelled within the figure.

Studies in treated IBD patients that measure T cell responses from the point of induction through to their persistence in immune memory are limited. A surge in the literature emerged following the widespread administration of novel COVID-19 vaccines in the general population, accompanied by reports of highly robust cellular and humoral responses induced by COVID-19 vaccination [49]. As mentioned, SARS-CoV-2-specific T cells detected both in the periphery and in tissues are linked to protection against COVID-19 [43,44]. Typically, peripheral T cell responses to vaccination are measured in venous blood samples drawn from donors, directly or after PBMC separation, by peptide antigen stimulation. T cells that recognize peptide antigens processed and displayed by blood APCs are then characterized according to the magnitude, kinetics, and functional profile of SARS-CoV-2-specific T cells in an infection-naïve patient. This review focuses on responses induced by mRNA vaccination, as these are often preferred and prevalent in IBD patients [50], as summarized in Figure 1.

### 3.1. Magnitude and Kinetics of Vaccine-Induced T Cells

Naïve CD4+ and CD8+ T cells that recognize SARS-CoV-2 antigens are induced by immunologic stimuli from COVID-19 mRNA vaccination, with each clone potentially undergoing a dramatic clonal expansion of up to 10^5^-fold. Peak magnitudes of SARS-CoV-2-specific T cell responses measured in the peripheral blood of healthy adults are achieved in a matter of weeks following a 2-dose priming series [51,52]. Reassuringly, robust responses in infliximab-treated IBD patients are achieved post vaccination, as the total Spike-specific CD4+ and CD8+ T cells quantified in isolated PBMCs 3 weeks after a single dose [53] and 2 weeks following two doses were comparable to healthy adults [54]. One study that instead relied on TCRβ sequencing, Spike-specific TCR annotation, and depth quantification in blood genomic DNA revealed that these parameters are increased in anti-TNF-treated IBD compared to untreated IBD patients [55]. Using fluorochrome-labeled peptide-MHC-I tetramer complexes that directly label antigen-specific CD8+ T cells without requiring stimulation, similar magnitudes of cellular frequencies and specificities were observed between anti-TNF-treated patients and healthy controls [56]. These findings hint that the stimuli introduced by mRNA vaccination in these individuals is sufficient to activate T cells, even when TNF is neutralized. This might imply that at least in the case of mRNA vaccination, TNF is inconsequential in the initial induction of these T cells, given that both the quantity and function of antigen-specific T cells remain intact. It should be noted that TNF is likely produced by CD14+ cells following the introduction of RNA–lipoprotein complexes, as suggested by the loss of TNF production with CD14+ cell depletion in vitro [57]. Another possibility is that the sequestration of TNF may be compensated for by the increased production of other pro-inflammatory cytokines that are yet to be defined.

More protracted studies looking into the longevity of these responses likewise reported sustained detection of SARS-CoV-2-specific responses. Our own study [58] examined vaccine-induced T cell responses by measuring IFN-γ and IL-2 directly in stimulated whole-blood samples from patients with IBD on various therapies, which offers the advantage of interrogating responses in the presence of therapeutic levels of immunomodulators [59], which is diluted out when peripheral blood mononuclear cells (PBMCs) are separated. Using a similar assay, we and others [60] noted that IBD patients demonstrate sustained T cell responses even 3 and 6 months after 2-dose vaccination. Uniquely, those on anti-TNF therapy displayed responses that were greater in magnitude than healthy controls, whether these were interrogated in whole blood or in PBMCs [58,61]. This is likely due to higher frequencies of IFN-γ producing Spike-specific T cells that persisted in the circulation [58]. Studies observing the longevity of vaccine-primed T cell responses beyond 6 months are limited, as they are likely less relevant given the increase in booster vaccine willingness [62], uptake [63], and the prevalence of SARS-CoV-2 breakthrough infections. It is important to point out that whether TNF inhibition directly alters the longevity of T cells induced by mRNA vaccination through co-stimulation/inhibition [64], indirectly by altering macrophage dynamics [65], or perhaps by reducing vaccine antigen clearance has yet to be determined.

### 3.2. Functional Profile

While the findings of improved cellular response magnitudes were notable, their implications were further heightened by the observation that, in contrast, humoral responses were deficient and declined rapidly. Several studies have consistently observed the finding of lower anti-Spike receptor binding domain (RBD) IgG antibody titers and poor serum neutralizing capacity. Even in patients who were labeled as anti-TNF-treated, the seropositivity rates of vaccine-induced antibodies were notably higher in patients with undetectable anti-TNF drug levels [66]. Moreover, these antibodies rapidly declined within 3 months in IBD patients on infliximab but not vedolizumab [67]. Such findings point towards the role of TNF in the formation and organization of germinal centers, as well as B cell maturation [68]. Moreover, T follicular helper cells (T_FH_) that aid B cell activation maintain high TNFR expression relative to other CD4+ subsets and have been shown to depend on TNF/NF-κB for their survival, as well as to promote T_FH_-B cell interactions to increase antibody production. These may be blocked by anti-TNF drugs, independent of naïve B cell activation [69]. Two studies assessed the frequencies of activated circulating T_FH_ (cT_FH_) in early post-vaccination samples of anti-TNF-treated IBD patients (1–2 weeks post 2-dose vaccination). Boland et al. reported lower overall Spike-specific cT_FH_ in both infliximab- and vedolizumab-treated patients despite having higher overall cT_FH_ relative to healthy controls [54]. Furthermore, Garner-Spitzer et al. found lower activated cT_FH_ mainly in patients treated with anti-TNF patients relative to those on anti-integrin drugs and demonstrated correlations between the activated cT_FH_ and both Spike S1-specific IgG and the frequency of S-specific memory B cells [70]. Booster (third-dose) vaccination assessed after 1–3 months was noted to exert similar degrees of activation of CD4/8+ T cells, as well as cT_FH_ subsets associated with further increased anti-Spike IgG titers in IBD patients; however, those on anti-TNF continue to exhibit lower vaccine-induced antibody titers than donors on other biologics [71], likewise seen in a separate study looking at responses 5 months post boost [72].

Beyond their roles in promoting T-dependent antibody responses, CD4+ T cells of varying subclasses may be polarized to produce distinct cytokine milieus in response to immunogenic stimuli. As in healthy individuals vaccinated with COVID-19 mRNA vaccines [51], T_H_1 responses, characterized by the production of IFN-γ, IL-2, and TNF-α, are likewise produced by T cells from IBD patients regardless of treatment following SARS-CoV-2 Spike peptide stimulation. The preceding literature on anti-TNF-treated patients, however, pointed towards evidence of the IL-10 polarization of circulating T cells following anti-TNF administration, with increases in IL-10 production observed in stimulated T cells following the initiation of anti-TNF [73,74]. Encouragingly, it has been suggested that the potential to modify classical T_H_1 T cell responses may be advantageous in the response to SARS-CoV-2 infection. It was recognized early that severe COVID-19 was associated with defective polyfunctional T cell responses [75], which in contrast is intact and robust in patients who control SARS-CoV-2 infection asymptomatically [76]. Indeed, the T cell response of individuals with hybrid SARS-CoV-2 immunity who demonstrate a robust immunity from re-infection [77,78] is characterized by the co-production of IFN-γ and IL-10 [79]. These observations are supported by animal studies demonstrating the role of simultaneous T cell secretion in effective and quiescent viral control [32,80,81]. Importantly, the finding that mRNA vaccination in IBD patients receiving TNF inhibitors leads to the activation of T cells with an IFN-γ/IL-2/IL-10 secretion profile indicates that comparable functional profiles could also arise in virus-specific T cells following SARS-CoV-2 infection. This may help clarify why SARS-CoV-2 infections tend to be mild in patients undergoing anti-TNF treatment [82,83,84].

One important consideration in the T cellular response of patients with immune-mediated inflammatory diseases (IMIDs), particularly IBD, is the potential to induce T_H_17 responses. Recent studies point towards a role of pathological T_H_17 polarization, given their enrichment in both intestinal and extraintestinal sites in active inflammation [85], which raises the question of whether vaccination in IBD induces cells polarized towards the T_H_17 spectrum. To the best of our knowledge, no vaccination studies in IBD focus on the induction of such responses. Instead, one study looked at IL-17A/IL-22 production by FluoroSpot assays of Spike peptide-pulsed PBMCs in patients with psoriasis undergoing various treatments after a single BNT162b2 mRNA vaccine dose, showing the low production of such cytokines in psoriasis patients undergoing anti-TNF therapy (including methotrexate and anti-IL-17 and anti-IL-23 therapies), similar to healthy controls [86].

## 4. Effects of Other Immunotherapies Used in IBD on Vaccine-Induced T Cell Responses

**Figure 2:**
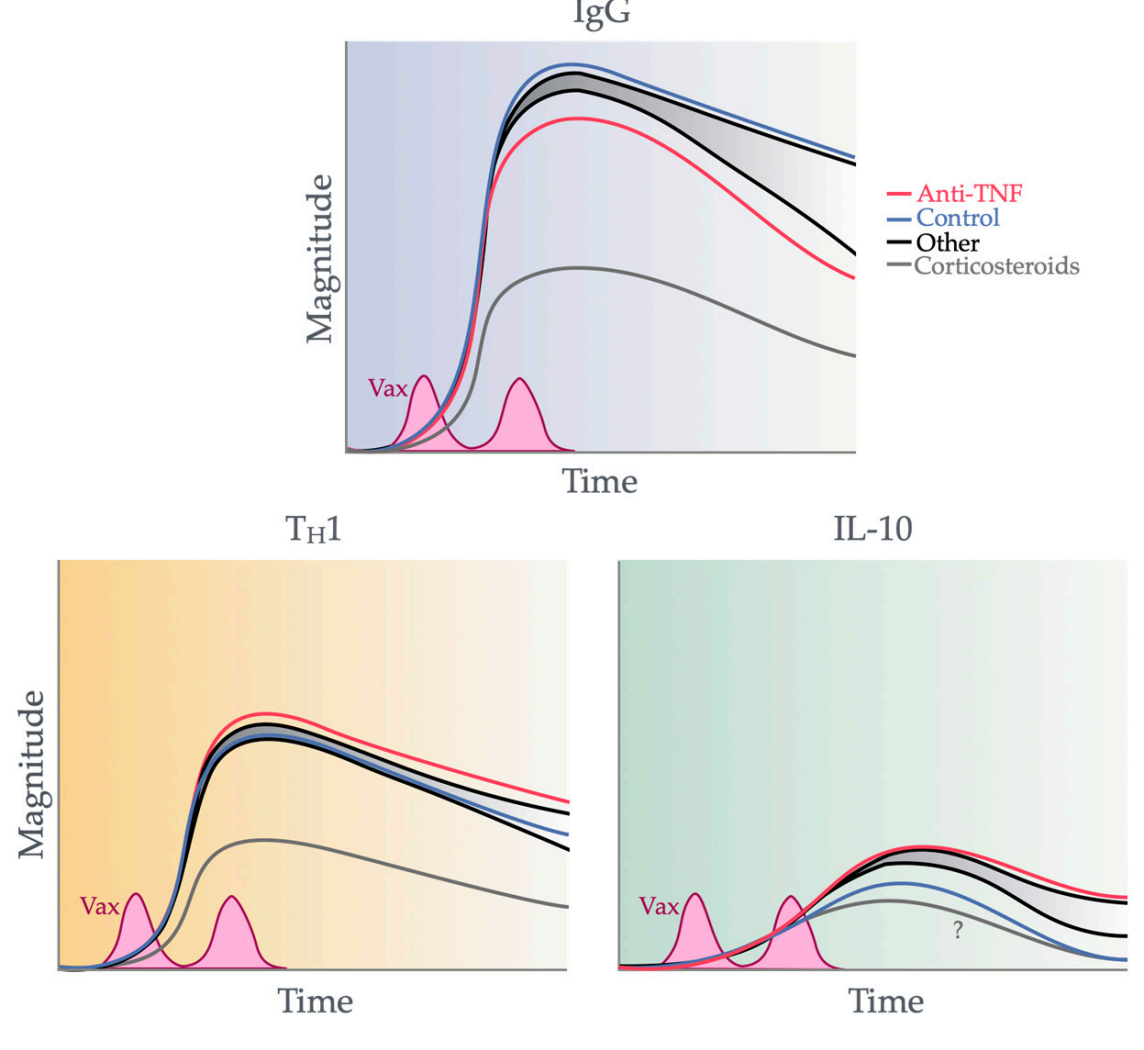
Impact of anti-inflammatory drugs on the magnitude of peripheral adaptive immune responses in patients with IBD induced by primary vaccination. Most vaccines induce humoral (IgG) and cellular responses (T_H_1 or potentially IL-10) to vaccine antigens. The vaccination of patients undergoing anti-TNF therapy induces poor IgG titers but intact or higher T_H_1/IL-10 cellular responses. Corticosteroids are associated with lower-magnitude induction of responses; however, their effects remain unknown for vaccine-induced IL-10 responses. The other anti-inflammatory drugs (non-anti-TNF biologics and antimetabolites) variably preserve humoral responses but mostly preserve vaccine-induced cellular responses.

Most studies assessing T cell responses induced by vaccination in IBD patients on immunotherapy have largely focused the effects of anti-TNF versus non-anti-TNF biologic therapies. Studies that do include patients on non-TNF biologics, including our own study, mainly consolidate them as a combined cohort, likely due to the lower prevalence of its usage. Importantly, non-anti-TNF-based therapies are better known to spare vaccine immunogenicity historically and are less linked to increased susceptibility to infection [87,88]. Here, we will explore the available evidence of the influence of systemic immunomodulators used in IBD other than anti-TNF biologics on the magnitude, function, and phenotype of vaccine-induced T cells (Figure 2).

### 4.1. Antimetabolite Drugs

Antimetabolite drugs such as methotrexate and azathioprine are widely used not only in IBD but also in other IMIDs. They work primarily by limiting purines and pyrimidines required for DNA synthesis, to which rapidly proliferating immune cells are highly sensitive. The usage of antimetabolite drugs is occasionally linked to poor seroconversion against seasonal influenza [89] or hepatitis B vaccines [90]. With COVID-19 mRNA vaccines, antimetabolite monotherapy utilization in patients with IBD or other IMIDs is not associated with reduced vaccine-induced antibody or T cell responses [58,60,86,91], while the rates of hospitalization or death remain low following vaccination [92,93]. More interesting, however, is the finding that the combined use of antimetabolites and anti-TNF inhibitors seems to exert a synergistic effect in reducing both vaccine-induced cellular and humoral responses and is often linked to poorer vaccine efficacy with more severe outcomes [92]. In our own study, while this inhibitory effect did not cause vaccine-induced cellular responses to significantly differ from those found in healthy controls, we found that this synergistic effect can already be observed in both early and late post-vaccination responses when compared to responses induced by those treated with anti-TNF therapy alone. It may be reasonable to speculate that the mechanism of increased memory responses generated in vaccinated donors on anti-TNF therapy alone crucially depends on cellular processes hindered by antimetabolites.

### 4.2. Corticosteroids

Corticosteroid drugs are among the most potent immunosuppressive drugs used in the induction of disease remission. Prolonged use, however, is associated with significant morbidity due to its metabolic and immunological side effects [94]. Thus, these drugs are used in short periods, which may explain the lack of dedicated studies in vaccinated IBD patients, most of which are performed on donors in remission with stable pharmaceutical regimens. Classically, they alter the immune response by inhibiting the NF-κB axis in a wide variety of cell types, impeding their activation in the face of an antigenic stimulus [95]. Concurrent corticosteroid usage in IBD patients undergoing COVID-19 vaccination was linked to a poor cellular and humoral responses to vaccination, as well as an increased risk of breakthrough infection or hospitalization [96,97], with conflicting findings in other studies potentially explained by the variability of timing and dosing of steroids used. Although few were involved in our own study, vaccinated patients on concurrent corticosteroid usage consistently demonstrated low-magnitude humoral and cellular Spike-specific responses.

### 4.3. Anti-Cytokine Biologics

Anti-cytokine biologics target the p40 subunit, a component of IL-12 and IL-23, preventing the interaction of these cytokines with their cognate receptors. Increased levels of both cytokines in inflammatory bowel disease primarily drives T_H_17 polarization and subsequent T_H_1-like potential that increases its potential to induce colitis [98]. Similarly to anti-TNF therapies, anti-p40 biologics have been labeled with theoretical risks of disseminated infections of M. tuberculosis, or even live-attenuated vaccines; however, these adverse events are rare [99]. Despite the inhibition of IL-12, which is known to drive the T_H_1 polarization of activated CD4+ T cells that have encountered vaccine-derived antigens, the production of IFN-γ and even higher levels of IL-2 were maintained in antigen-stimulated samples from donors on ustekinumab [58]. Meanwhile, seroconversion following COVID-19 mRNA administration remained intact and similar to healthy controls in the same IBD donors demonstrating robust T cell responses [58] and in larger multicenter studies [91].

### 4.4. Anti-Integrin Biologics

In contrast to anti-TNF, or even anti-p40 therapies, anti-integrin antibodies such as vedolizumab or etrolizumab are less implicated in systemic immunosuppression. This is likely due to their gut-specific effect, which is to inhibit leukocytic migration into Peyer’s patches and the lamina propria by the blockade of integrins (α4β7 in vedolizumab or αEβ7 in etrolizumab) that confer interactions with intestinal MAdCAM-1 [100]. Interestingly, however, while these effects were previously thought to largely affect the migration of T cell populations in the gut, a study by Zeissig et al. found minimal changes in the T cellular repertoire of the circulation and intestines following vedolizumab treatment, and, rather, found more significant alterations in intestinal macrophage populations [101]. Nevertheless, these effects seem to establish an immune system that simulates the extraintestinal cytokine milieu of healthy individuals. Indeed, both antibody and T cell responses induced by COVID-19 vaccination in vedolizumab-treated individuals are comparable to healthy controls [58,102].

## 5. TNF Blockade and Pre-Existing Memory T Cell Responses

**Figure 3:**
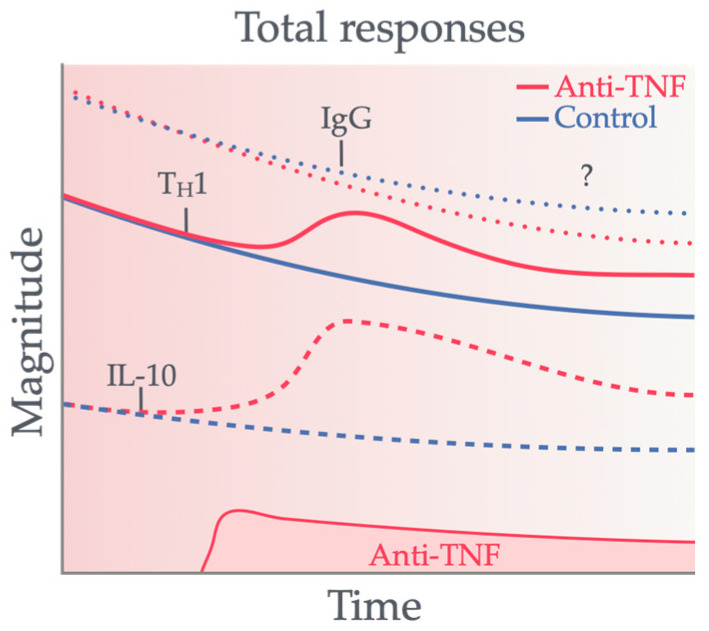
Impact of anti-TNF drugs on the magnitude and profile of global adaptive immune responses. Anti-TNF drugs are associated with increased magnitudes in cellular responses; however, their effects on total IgG are unknown. The negative effects of anti-TNF therapy on germinal center B cells and long-lived plasma cells may cause an overall decrease in total IgG over time [108]. The line styles represent the different types of peripheral immune responses measured, as labelled within the figure.

As alluded to in prior sections, the administration of anti-TNF drugs also impacts the responses of pre-established memory T cells (Figure 3). TNF is a pleiotropic cytokine that mainly exerts immunologic function. As a therapeutic target in IBD, local pro-inflammatory TNF, derived predominantly from intestinal dendritic cells and monocytes [103] but also from T cells [104], is directed for neutralization. This contributes to colitis by activating inflammation, reducing gut barrier integrity, and increasing pro-inflammatory cell influx. Meanwhile, within the circulation, the prolonged exposure of T cells to pro-inflammatory cytokines such as TNF in vitro was shown to impair their activation and effector function. A recent study by Globig et al. found that a subset of CD8+ T cells displaying an exhausted CD39+ PD-1+ signature were enriched in active Crohn’s disease when compared to remission, which quantitatively correlates with disease activity measured by the Harvey–Bradshaw index [105].

Studies in circulating T cells following anti-TNF treatment demonstrated an uptrend in several facets of T cell effector function, such as proliferation and cytokine production. This is evidenced by an increased index of expansion following anti-TNF treatment for active rheumatoid arthritis [106] or an increase in T_H_1/2/17 cytokines produced in IBD and psoriasis [107]. Whether these findings are due to an increased proportion of polarized effector T cells or the increased functionality of individual T cells remains unclear.

## 6. Vaccine-Induced T Cell-Mediated Immunity and Protection Against Infectious Disease

Although several favorable properties of SARS-CoV-2-specific T cells raised by COVID-19 vaccination in anti-TNF-treated IBD patients were identified, their independent impact on protection from disease is difficult to confidently ascertain. One important aspect to consider is the direct role of anti-TNF drugs in SARS-CoV-2 infections, which was once considered a direct therapeutic [109]. Meanwhile, the residual number of vaccine-induced antibodies mounted by donors on anti-TNF may still potentially exert partially beneficial roles in reducing SARS-CoV-2 burden. Even IBD activity may confound analyses of vaccine-induced protection, as active colitis was associated with negative COVID-19 outcomes [110], although most vaccination studies involve IBD patients in disease remission.

Vaccination status in patients with IBD, relative to healthy controls, is associated with low rates of COVID-19-related outcomes, including breakthrough infection [111], hospitalization [92,112], and death [92], compared to no vaccination. Findings in Israel (epi-IIRN) stratified IBD patients according to immunomodulator, and found no difference in infection from vaccinated healthy controls, even in patients on anti-TNF [113]. Post-vaccination all-cause hospitalization was also reported to be reduced in one study [114]. In the PREVENT-COVID study cohort, comprising a majority of IBD patients on anti-TNF therapy, hospitalizations were rare even in those who experienced vaccine breakthrough infections [115]. Beyond IBD status, a Danish study found hospitalization and death as rare post-vaccination outcomes in IMID patients on anti-TNF therapy as a class [116]. A third-dose vaccination study comparing IBD patients on infliximab or vedolizumab found that the severe outcomes of hospitalization and death remained low, in spite of high breakthrough infection rates during the Omicron variant wave [117].

## 7. Conclusions

The findings reviewed highlight the complexity of immune responses in IBD patients treated with anti-TNF biologics, particularly in the context of vaccination. The emerging evidence of robust vaccine-induced T cell responses, despite diminished humoral responses, suggests that the immunological landscape in these patients is more nuanced than previously understood. These insights challenge the generalized view of anti-TNF therapy as predominantly immunosuppressive and highlight the need for a careful evaluation of how these treatments interact with the adaptive immune system. Indeed, varying immune-related conditions alter the host peripheral immune system themselves [118] in ways that only further evaluation of drug–disease interactions would reveal.

The most obvious clinical implication would be that the magnitude and properties of cellular adaptive responses raised by vaccines may be used to assess their immunogenicity, particularly in patients who poorly seroconvert. While the specific T cell correlates of protection are yet to be definitively outlined for several infectious diseases, recent developments in SARS-CoV-2 have identified candidates that may be considered [119,120]. This area is rapidly evolving with developments in both vaccination strategies and T cell assays that may eventually be implemented [121].

Moreover, the recognition of the functional profiles of these T cells, including the possibility of skewing the cytokine milieu, provides a promising area for broader vaccine development and therapeutic strategies. This could involve targeting the enhancement of T cell responses in or beyond IBD patients or tailoring vaccines to bolster these cellular defenses. Meanwhile, such response profiles may not be desirable and may even be linked to detrimental outcomes, such as in tuberculosis [122].

As these findings emerged within the context of SARS-CoV-2, they require careful evaluation when applied within the context of different vaccine-preventable diseases. The introduction of novel mRNA vaccines has inevitably created a promising tool for inducing robust T cell responses that may be directed against infectious diseases of concern in this population. Vaccination strategies may be implemented to maximize or minimize the effects of immunomodulatory therapy on vaccine-induced responses.

Many unanswered questions remain. On the subject of vaccine-induced responses, it remains uncertain whether this phenotype of long-lived, balanced inflammatory response persists over time or amidst repeated antigen encounters in the form of booster vaccination or breakthrough (re)infection. As the COVID-19 pandemic has receded, and SARS-CoV-2 has entered endemic spread, it has become difficult to track the longevity of vaccine-induced responses in genuinely unexposed individuals. Instead, the focus should shift towards assessing the functional phenotype of vaccine-induced T cells in individuals who have encountered repeated antigen exposures, especially when hybrid immunity has become better characterized in healthy individuals. Moreover, the interaction between anti-TNF drugs and the capacity to develop immunity at mucocutaneous sites following breakthrough infection may be an important aspect to tackle, in and beyond the context of SARS-CoV-2, as barrier site immunity has been shown to develop in individuals with SARS-CoV-2 hybrid immunity [34,35] and is perhaps linked to improved outcomes against pathogens.

Another open question concerns the influence of chronic anti-TNF administration on the stability of the identified properties and whether a change in the biologic class used would alter their properties. This may be approached via studies conducted in a cohort of patients who have switched or are planning to switch biologics from anti-TNF to non-anti-TNF biologics and vice versa.

The evolving landscape of therapies, as well as pharmacological strategies in IBD, may further add complexity to the study of vaccine-induced responses in this population. However, the trend towards the pharmacological targeting of gut-specific pathways with less off-target effects suggests that the influence of therapies may play less influential roles in the development of adaptive immune responses. Instead, this may aid in revealing the impact of disease control on systemic adaptive immunity and perhaps shed some light on its interactions with localized gut events.

Importantly, the implications of therapies used in IBD on the development of adaptive immunity may likewise exert similar effects when used for patients with other inflammatory diseases. Future studies that investigate whether the immunological phenomena incited by anti-TNF therapy in IBD likewise apply in other conditions could investigate these cohorts in parallel and in more depth.

In conclusion, while the current evidence provides valuable insights, it also raises important questions that warrant further investigation. Continued research into the interaction between anti-TNF therapy and vaccine-induced T cell responses will be essential in advancing our understanding of immune protection in IBD and guiding future clinical practices.

## References

[1] Wang R., Li Z., Liu S., Zhang D. (2023). Global, Regional and National Burden of Inflammatory Bowel Disease in 204 Countries and Territories from 1990 to 2019: A Systematic Analysis Based on the Global Burden of Disease Study 2019. BMJ Open.

[2] Kaplan G.G. (2015). The Global Burden of IBD: From 2015 to 2025. Nat. Rev. Gastroenterol. Hepatol..

[3] Cai Z., Wang S., Li J. (2021). Treatment of Inflammatory Bowel Disease: A Comprehensive Review. Front. Med..

[4] Berg D.R., Colombel J.-F., Ungaro R. (2019). The Role of Early Biologic Therapy in Inflammatory Bowel Disease. Inflamm. Bowel Dis..

[5] Neurath M.F., Travis S.P.L. (2012). Mucosal Healing in Inflammatory Bowel Diseases: A Systematic Review. Gut.

[6] Keane J., Gershon S., Wise R.P., Mirabile-Levens E., Kasznica J., Schwieterman W.D., Siegel J.N., Braun M.M. (2001). Tuberculosis Associated with Infliximab, a Tumor Necrosis Factor Alpha-Neutralizing Agent. N. Engl. J. Med..

[7] Millonig G., Kern M., Ludwiczek O., Nachbaur K., Vogel W. (2006). Subfulminant Hepatitis B after Infliximab in Crohn’s Disease: Need for HBV-Screening?. World J. Gastroenterol..

[8] Toruner M., Loftus E.V., Harmsen W.S., Zinsmeister A.R., Orenstein R., Sandborn W.J., Colombel J.-F., Egan L.J. (2008). Risk Factors for Opportunistic Infections in Patients with Inflammatory Bowel Disease. Gastroenterology.

[9] Lamb C.A., Kennedy N.A., Raine T., Hendy P.A., Smith P.J., Limdi J.K., Hayee B., Lomer M.C.E., Parkes G.C., Selinger C. (2019). British Society of Gastroenterology Consensus Guidelines on the Management of Inflammatory Bowel Disease in Adults. Gut.

[10] Plotkin S.A. (2008). Vaccines: Correlates of Vaccine-Induced Immunity. Clin. Infect. Dis. Off. Publ. Infect. Dis. Soc. Am..

[11] Marín A.C., Gisbert J.P., Chaparro M. (2015). Immunogenicity and Mechanisms Impairing the Response to Vaccines in Inflammatory Bowel Disease. World J. Gastroenterol. WJG.

[12] Salinas G.F., De Rycke L., Barendregt B., Paramarta J.E., Hreggvidsdottir H., Cantaert T., van der Burg M., Tak P.P., Baeten D. (2013). Anti-TNF Treatment Blocks the Induction of T Cell-Dependent Humoral Responses. Ann. Rheum. Dis..

[13] Bertoletti A., Le Bert N., Tan A.T. (2022). SARS-CoV-2-Specific T Cells in the Changing Landscape of the COVID-19 Pandemic. Immunity.

[14] Zinkernagel R.M., Doherty P.C. (1974). Restriction of in Vitro T Cell-Mediated Cytotoxicity in Lymphocytic Choriomeningitis within a Syngeneic or Semiallogeneic System. Nature.

[15] Goulmy E., Termijtelen A., Bradley B.A., Van Rood J.J. (1977). Y-Antigen Killing by T Cells of Women Is Restricted by HLA. Nature.

[16] Sette A., Crotty S. (2021). Adaptive Immunity to SARS-CoV-2 and COVID-19. Cell.

[17] Bergholtz B.O., Thorsby E. (1977). Macrophage-Dependent Response of Immune Human T Lymphocytes to PPD In Vitro Influence of HLA-D Histocompatibility. Scand. J. Immunol..

[18] Tay R.E., Richardson E.K., Toh H.C. (2021). Revisiting the Role of CD4+ T Cells in Cancer Immunotherapy—New Insights into Old Paradigms. Cancer Gene Ther..

[19] Tuzlak S., Dejean A.S., Iannacone M., Quintana F.J., Waisman A., Ginhoux F., Korn T., Becher B. (2021). Repositioning TH Cell Polarization from Single Cytokines to Complex Help. Nat. Immunol..

[20] Agudo J., Ruzo A., Park E.S., Sweeney R., Kana V., Wu M., Zhao Y., Egli D., Merad M., Brown B.D. (2015). JEDI T-Cells Enable Targeted Cell Depletion and Investigation of T-Cell Interactions with Virtually Any Cell Population. Nat. Biotechnol..

[21] Robinson H.L., Amara R.R. (2005). T Cell Vaccines for Microbial Infections. Nat. Med..

[22] Wong M.T., Ong D.E.H., Lim F.S.H., Teng K.W.W., McGovern N., Narayanan S., Ho W.Q., Cerny D., Tan H.K.K., Anicete R. (2016). A High-Dimensional Atlas of Human T Cell Diversity Reveals Tissue-Specific Trafficking and Cytokine Signatures. Immunity.

[23] Sender R., Weiss Y., Navon Y., Milo I., Azulay N., Keren L., Fuchs S., Ben-Zvi D., Noor E., Milo R. (2023). The Total Mass, Number, and Distribution of Immune Cells in the Human Body. Proc. Natl. Acad. Sci. USA.

[24] Steinert E.M., Schenkel J.M., Fraser K.A., Beura L.K., Manlove L.S., Igyártó B.Z., Southern P.J., Masopust D. (2015). Quantifying Memory CD8 T Cells Reveals Regionalization of Immunosurveillance. Cell.

[25] Farber D.L. (2021). Tissues, Not Blood, Are Where Immune Cells Function. Nature.

[26] Beura L.K., Fares-Frederickson N.J., Steinert E.M., Scott M.C., Thompson E.A., Fraser K.A., Schenkel J.M., Vezys V., Masopust D. (2019). CD4+ Resident Memory T Cells Dominate Immunosurveillance and Orchestrate Local Recall Responses. J. Exp. Med..

[27] Turner D.L., Farber D.L. (2014). Mucosal Resident Memory CD4 T Cells in Protection and Immunopathology. Front. Immunol..

[28] Mackay L.K., Stock A.T., Ma J.Z., Jones C.M., Kent S.J., Mueller S.N., Heath W.R., Carbone F.R., Gebhardt T. (2012). Long-Lived Epithelial Immunity by Tissue-Resident Memory T (TRM) Cells in the Absence of Persisting Local Antigen Presentation. Proc. Natl. Acad. Sci. USA.

[29] Zheng M.Z.M., Wakim L.M. (2022). Tissue Resident Memory T Cells in the Respiratory Tract. Mucosal Immunol..

[30] Sridhar S. (2016). Heterosubtypic T-Cell Immunity to Influenza in Humans: Challenges for Universal T-Cell Influenza Vaccines. Front. Immunol..

[31] Sridhar S., Begom S., Bermingham A., Hoschler K., Adamson W., Carman W., Bean T., Barclay W., Deeks J.J., Lalvani A. (2013). Cellular Immune Correlates of Protection against Symptomatic Pandemic Influenza. Nat. Med..

[32] Zhao J., Zhao J., Mangalam A.K., Channappanavar R., Fett C., Meyerholz D.K., Agnihothram S., Baric R.S., David C.S., Perlman S. (2016). Airway Memory CD4(+) T Cells Mediate Protective Immunity against Emerging Respiratory Coronaviruses. Immunity.

[33] Wu T., Hu Y., Lee Y.-T., Bouchard K.R., Benechet A., Khanna K., Cauley L.S. (2014). Lung-Resident Memory CD8 T Cells (TRM) Are Indispensable for Optimal Cross-Protection against Pulmonary Virus Infection. J. Leukoc. Biol..

[34] Lim J.M.E., Tan A.T., Le Bert N., Hang S.K., Low J.G.H., Bertoletti A. (2022). SARS-CoV-2 Breakthrough Infection in Vaccinees Induces Virus-Specific Nasal-Resident CD8+ and CD4+ T Cells of Broad Specificity. J. Exp. Med..

[35] Mitsi E., Diniz M.O., Reiné J., Collins A.M., Robinson R.E., Hyder-Wright A., Farrar M., Liatsikos K., Hamilton J., Onyema O. (2023). Respiratory Mucosal Immune Memory to SARS-CoV-2 after Infection and Vaccination. Nat. Commun..

[36] Tang J., Zeng C., Cox T.M., Li C., Son Y.M., Cheon I.S., Wu Y., Behl S., Taylor J.J., Chakaraborty R. (2022). Respiratory Mucosal Immunity against SARS-CoV-2 after mRNA Vaccination. Sci. Immunol..

[37] Fletcher H.A., Snowden M.A., Landry B., Rida W., Satti I., Harris S.A., Matsumiya M., Tanner R., O’Shea M.K., Dheenadhayalan V. (2016). T-Cell Activation Is an Immune Correlate of Risk in BCG Vaccinated Infants. Nat. Commun..

[38] Mittrücker H.-W., Steinhoff U., Köhler A., Krause M., Lazar D., Mex P., Miekley D., Kaufmann S.H.E. (2007). Poor Correlation between BCG Vaccination-Induced T Cell Responses and Protection against Tuberculosis. Proc. Natl. Acad. Sci. USA.

[39] Mettelman R.C., Souquette A., Van de Velde L.-A., Vegesana K., Allen E.K., Kackos C.M., Trifkovic S., DeBeauchamp J., Wilson T.L., St. James D.G. (2023). Baseline Innate and T Cell Populations Are Correlates of Protection against Symptomatic Influenza Virus Infection Independent of Serology. Nat. Immunol..

[40] Carabelli A.M., Peacock T.P., Thorne L.G., Harvey W.T., Hughes J., De Silva T.I., Peacock S.J., Barclay W.S., De Silva T.I., COVID-19 Genomics UK Consortium (2023). SARS-CoV-2 Variant Biology: Immune Escape, Transmission and Fitness. Nat. Rev. Microbiol..

[41] Collie S., Champion J., Moultrie H., Bekker L.-G., Gray G. (2022). Effectiveness of BNT162b2 Vaccine against Omicron Variant in South Africa. N. Engl. J. Med..

[42] Kirsebom F.C.M., Andrews N., Stowe J., Toffa S., Sachdeva R., Gallagher E., Groves N., O’Connell A.M., Chand M., Ramsay M. (2022). COVID-19 Vaccine Effectiveness against the Omicron (BA.2) Variant in England. Lancet Infect. Dis..

[43] McMahan K., Yu J., Mercado N.B., Loos C., Tostanoski L.H., Chandrashekar A., Liu J., Peter L., Atyeo C., Zhu A. (2021). Correlates of Protection against SARS-CoV-2 in Rhesus Macaques. Nature.

[44] Koutsakos M., Reynaldi A., Lee W.S., Nguyen J., Amarasena T., Taiaroa G., Kinsella P., Liew K.C., Tran T., Kent H.E. (2023). SARS-CoV-2 Breakthrough Infection Induces Rapid Memory and de Novo T Cell Responses. Immunity.

[45] Painter M.M., Johnston T.S., Lundgreen K.A., Santos J.J.S., Qin J.S., Goel R.R., Apostolidis S.A., Mathew D., Fulmer B., Williams J.C. (2023). Prior Vaccination Promotes Early Activation of Memory T Cells and Enhances Immune Responses during SARS-CoV-2 Breakthrough Infection. Nat. Immunol..

[46] Tan A.T., Linster M., Tan C.W., Le Bert N., Chia W.N., Kunasegaran K., Zhuang Y., Tham C.Y.L., Chia A., Smith G.J.D. (2021). Early Induction of Functional SARS-CoV-2-Specific T Cells Associates with Rapid Viral Clearance and Mild Disease in COVID-19 Patients. Cell Rep..

[47] Swadling L., Diniz M.O., Schmidt N.M., Amin O.E., Chandran A., Shaw E., Pade C., Gibbons J.M., Le Bert N., Tan A.T. (2022). Pre-Existing Polymerase-Specific T Cells Expand in Abortive Seronegative SARS-CoV-2. Nature.

[48] Augusto D.G., Murdolo L.D., Chatzileontiadou D.S.M., Sabatino J.J., Yusufali T., Peyser N.D., Butcher X., Kizer K., Guthrie K., Murray V.W. (2023). A Common Allele of HLA Is Associated with Asymptomatic SARS-CoV-2 Infection. Nature.

[49] Zhang Z., Mateus J., Coelho C.H., Dan J.M., Moderbacher C.R., Gálvez R.I., Cortes F.H., Grifoni A., Tarke A., Chang J. (2022). Humoral and Cellular Immune Memory to Four COVID-19 Vaccines. Cell.

[50] Kucharzik T., Ellul P., Greuter T., Rahier J.F., Verstockt B., Abreu C., Albuquerque A., Allocca M., Esteve M., Farraye F.A. (2021). ECCO Guidelines on the Prevention, Diagnosis, and Management of Infections in Inflammatory Bowel Disease. J. Crohns Colitis.

[51] Sahin U., Muik A., Vogler I., Derhovanessian E., Kranz L.M., Vormehr M., Quandt J., Bidmon N., Ulges A., Baum A. (2021). BNT162b2 Vaccine Induces Neutralizing Antibodies and Poly-Specific T Cells in Humans. Nature.

[52] Oberhardt V., Luxenburger H., Kemming J., Schulien I., Ciminski K., Giese S., Csernalabics B., Lang-Meli J., Janowska I., Staniek J. (2021). Rapid and Stable Mobilization of CD8+ T Cells by SARS-CoV-2 mRNA Vaccine. Nature.

[53] Reuken P.A., Andreas N., Grunert P.C., Glöckner S., Kamradt T., Stallmach A. (2022). T Cell Response After SARS-CoV-2 Vaccination in Immunocompromised Patients with Inflammatory Bowel Disease. J. Crohns Colitis.

[54] Boland B.S., Goodwin B., Zhang Z., Bloom N., Kato Y., Neill J., Le H., Tysl T., Collins A.E., Dulai P.S. (2022). Preserved SARS-CoV-2 Vaccine Cell-Mediated Immunogenicity in Patients with Inflammatory Bowel Disease on Immune-Modulating Therapies. Clin. Transl. Gastroenterol..

[55] Li D., Xu A., Mengesha E., Elyanow R., Gittelman R.M., Chapman H., Prostko J.C., Frias E.C., Stewart J.L., Pozdnyakova V. (2022). The T-Cell Response to SARS-CoV-2 Vaccination in Inflammatory Bowel Disease Is Augmented with Anti-TNF Therapy. Inflamm. Bowel Dis..

[56] van den Dijssel J., Duurland M.C., Konijn V.A., Kummer L.Y., Hagen R.R., Kuijper L.H., Wieske L., van Dam K.P., Stalman E.W., Steenhuis M. (2024). mRNA-1273 Vaccinated Inflammatory Bowel Disease Patients Receiving TNF Inhibitors Develop Broad and Robust SARS-CoV-2-Specific CD8+ T Cell Responses. J. Autoimmun..

[57] Tahtinen S., Tong A.-J., Himmels P., Oh J., Paler-Martinez A., Kim L., Wichner S., Oei Y., McCarron M.J., Freund E.C. (2022). IL-1 and IL-1ra Are Key Regulators of the Inflammatory Response to RNA Vaccines. Nat. Immunol..

[58] Qui M., Le Bert N., Chan W.P.W., Tan M., Hang S.K., Hariharaputran S., Sim J.X.Y., Low J.G.H., Ng W., Wan W.Y. (2022). Favorable Vaccine-Induced SARS-CoV-2 Specific T Cell Response Profile in Patients Undergoing Immune-Modifying Therapies. J. Clin. Investig..

[59] Tan A.T., Le Bert N., Bertoletti A. (2021). Difference in Sensitivity between SARS-CoV-2-Specific T Cell Assays in Patients with Underlying Conditions. Reply. J. Clin. Investig..

[60] Vollenberg R., Tepasse P.-R., Lorentzen E., Nowacki T.M. (2022). Impaired Humoral Immunity with Concomitant Preserved T Cell Reactivity in IBD Patients on Treatment with Infliximab 6 Month after Vaccination with the SARS-CoV-2 mRNA Vaccine BNT162b2: A Pilot Study. J. Pers. Med..

[61] Caldera F., Farraye F.A., Necela B.M., Cogen D., Saha S., Wald A., Daoud N.D., Chun K., Grimes I., Lutz M. (2023). Higher Cell-Mediated Immune Responses in Patients with Inflammatory Bowel Disease on Anti-TNF Therapy After COVID-19 Vaccination. Inflamm. Bowel Dis..

[62] Shehab M., Alrashed F., Alfadhli A. (2022). COVID-19 Vaccine Booster Dose Willingness among Patients with Inflammatory Bowel Disease on Infliximab and Vedolizumab: A Cross-Sectional Study. Vaccines.

[63] Wellens J., Brann S., Adams A., Marlow L., Lindsay J.O., Satsangi J.J. (2022). Determinants of Uptake of a Third Dose of SARS-CoV-2 Vaccines in Patients with Inflammatory Bowel Disease. Lancet Gastroenterol. Hepatol..

[64] Ward-Kavanagh L.K., Lin W.W., Šedý J.R., Ware C.F. (2016). The TNF Receptor Superfamily in Co-Stimulating and Co-Inhibitory Responses. Immunity.

[65] Koelink P.J., Bloemendaal F.M., Li B., Westera L., Vogels E.W.M., van Roest M., Gloudemans A.K., van ’t Wout A.B., Korf H., Vermeire S. (2020). Anti-TNF Therapy in IBD Exerts Its Therapeutic Effect through Macrophage IL-10 Signalling. Gut.

[66] Chanchlani N., Lin S., Chee D., Hamilton B., Nice R., Arkir Z., Bewshea C., Cipriano B., Derikx L.A.A.P., Dunlop A. (2022). Adalimumab and Infliximab Impair SARS-CoV-2 Antibody Responses: Results from a Therapeutic Drug Monitoring Study in 11 422 Biologic-Treated Patients. J. Crohns Colitis.

[67] Kennedy N.A., Lin S., Goodhand J.R., Chanchlani N., Hamilton B., Bewshea C., Nice R., Chee D., Cummings J.F., Fraser A. (2021). Infliximab Is Associated with Attenuated Immunogenicity to BNT162b2 and ChAdOx1 nCoV-19 SARS-CoV-2 Vaccines in Patients with IBD. Gut.

[68] Pasparakis M., Alexopoulou L., Episkopou V., Kollias G. (1996). Immune and Inflammatory Responses in TNF Alpha-Deficient Mice: A Critical Requirement for TNF Alpha in the Formation of Primary B Cell Follicles, Follicular Dendritic Cell Networks and Germinal Centers, and in the Maturation of the Humoral Immune Response. J. Exp. Med..

[69] Herati R.S., Silva L.V., Vella L.A., Muselman A., Alanio C., Bengsch B., Kurupati R.K., Kannan S., Manne S., Kossenkov A.V. (2021). Vaccine-Induced ICOS+CD38+ Circulating Tfh Are Sensitive Biosensors of Age-Related Changes in Inflammatory Pathways. Cell Rep. Med..

[70] Garner-Spitzer E., Wagner A., Gudipati V., Schoetta A.-M., Orola-Taus M., Kundi M., Kunert R., Mayrhofer P., Huppa J.B., Stockinger H. (2023). Lower Magnitude and Faster Waning of Antibody Responses to SARS-CoV-2 Vaccination in Anti-TNF-α-Treated IBD Patients Are Linked to Lack of Activation and Expansion of cTfh1 Cells and Impaired B Memory Cell Formation. EBioMedicine.

[71] Zhang E., Nguyen T.H.O., Allen L.F., Kedzierski L., Rowntree L.C., Chang S.Y., Zhang W., Habel J.R., Foo I.J., Menon T. (2024). Robust SARS-CoV-2 Antibody and T Cell Immunity Following Three COVID-19 Vaccine Doses in Inflammatory Bowel Disease Patients Receiving Anti-TNF or Alternative Treatments. Gut.

[72] Pavia G., Spagnuolo R., Quirino A., Marascio N., Giancotti A., Simeone S., Cosco C., Tino E., Carrabetta F., Di Gennaro G. (2023). COVID-19 Vaccine Booster Shot Preserves T Cells Immune Response Based on Interferon-Gamma Release Assay in Inflammatory Bowel Disease (IBD) Patients on Anti-TNFα Treatment. Vaccines.

[73] Evans H.G., Roostalu U., Walter G.J., Gullick N.J., Frederiksen K.S., Roberts C.A., Sumner J., Baeten D.L., Gerwien J.G., Cope A.P. (2014). TNF-α Blockade Induces IL-10 Expression in Human CD4+ T Cells. Nat. Commun..

[74] Roberts C.A., Durham L.E., Fleskens V., Evans H.G., Taams L.S. (2017). TNF Blockade Maintains an IL-10+ Phenotype in Human Effector CD4+ and CD8+ T Cells. Front. Immunol..

[75] Chauss D., Freiwald T., McGregor R., Yan B., Wang L., Nova-Lamperti E., Kumar D., Zhang Z., Teague H., West E.E. (2022). Autocrine Vitamin D Signaling Switches off Pro-Inflammatory Programs of TH1 Cells. Nat. Immunol..

[76] Le Bert N., Clapham H.E., Tan A.T., Chia W.N., Tham C.Y.L., Lim J.M., Kunasegaran K., Tan L.W.L., Dutertre C.-A., Shankar N. (2021). Highly Functional Virus-Specific Cellular Immune Response in Asymptomatic SARS-CoV-2 Infection. J. Exp. Med..

[77] Reynolds C.J., Gibbons J.M., Pade C., Lin K.-M., Sandoval D.M., Pieper F., Butler D.K., Liu S., Otter A.D., Joy G. (2022). Heterologous Infection and Vaccination Shapes Immunity against SARS-CoV-2 Variants. Science.

[78] Abu-Raddad L.J., Chemaitelly H., Ayoub H.H., Yassine H.M., Benslimane F.M., Al Khatib H.A., Tang P., Hasan M.R., Coyle P., Al Kanaani Z. (2021). Association of Prior SARS-CoV-2 Infection with Risk of Breakthrough Infection Following mRNA Vaccination in Qatar. JAMA.

[79] Rodda L.B., Netland J., Shehata L., Pruner K.B., Morawski P.A., Thouvenel C.D., Takehara K.K., Eggenberger J., Hemann E.A., Waterman H.R. (2021). Functional SARS-CoV-2-Specific Immune Memory Persists after Mild COVID-19. Cell.

[80] Zhuang Z., Lai X., Sun J., Chen Z., Zhang Z., Dai J., Liu D., Li Y., Li F., Wang Y. (2021). Mapping and Role of T Cell Response in SARS-CoV-2–Infected Mice. J. Exp. Med..

[81] Sun J., Madan R., Karp C.L., Braciale T.J. (2009). Effector T Cells Control Lung Inflammation during Acute Influenza Virus Infection by Producing IL-10. Nat. Med..

[82] Salesi M., Shojaie B., Farajzadegan Z., Salesi N., Mohammadi E. (2021). TNF-α Blockers Showed Prophylactic Effects in Preventing COVID-19 in Patients with Rheumatoid Arthritis and Seronegative Spondyloarthropathies: A Case–Control Study. Rheumatol. Ther..

[83] Haberman R., Axelrad J., Chen A., Castillo R., Yan D., Izmirly P., Neimann A., Adhikari S., Hudesman D., Scher J.U. (2020). COVID-19 in Immune-Mediated Inflammatory Diseases—Case Series from New York. N. Engl. J. Med..

[84] Chappell H., Patel R., Driessens C., Tarr A.W., Irving W.L., Tighe P.J., Jackson H.J., Harvey-Cowlishaw T., Mills L., Shaunak M. (2022). Immunocompromised Children and Young People Are at No Increased Risk of Severe COVID-19. J. Infect..

[85] Chen L., Ruan G., Cheng Y., Yi A., Chen D., Wei Y. (2023). The Role of Th17 Cells in Inflammatory Bowel Disease and the Research Progress. Front. Immunol..

[86] Mahil S.K., Bechman K., Raharja A., Domingo-Vila C., Baudry D., Brown M.A., Cope A.P., Dasandi T., Graham C., Lechmere T. (2021). The Effect of Methotrexate and Targeted Immunosuppression on Humoral and Cellular Immune Responses to the COVID-19 Vaccine BNT162b2: A Cohort Study. Lancet Rheumatol..

[87] Singh S., Kim J., Luo J., Paul P., Rudrapatna V., Park S., Zheng K., Syal G., Ha C., Fleshner P. (2023). Comparative Safety and Effectiveness of Biologic Therapy for Crohn’s Disease: A CA-IBD Cohort Study. Clin. Gastroenterol. Hepatol..

[88] Chaaban L., Huang J., Melia J. (2024). Safety of Anti-Tumor Necrosis Factor Agents Compared to Ustekinumab And Vedolizumab in Elderly Patients with Ulcera-Tive Colitis. Inflamm. Bowel Dis..

[89] Cullen G., Bader C., Korzenik J.R., Sands B.E. (2012). Serological Response to the 2009 H1N1 Influenza Vaccination in Patients with Inflammatory Bowel Disease. Gut.

[90] Shirai S., Hara M., Sakata Y., Tsuruoka N., Yamamoto K., Shimoda R., Gomi Y., Yoshii H., Fujimoto K., Iwakiri R. (2018). Immunogenicity of Quadrivalent Influenza Vaccine for Patients with Inflammatory Bowel Disease Undergoing Immunosuppressive Therapy. Inflamm. Bowel Dis..

[91] Alexander J.L., Kennedy N.A., Ibraheim H., Anandabaskaran S., Saifuddin A., Castro Seoane R., Liu Z., Nice R., Bewshea C., D’Mello A. (2022). COVID-19 Vaccine-Induced Antibody Responses in Immunosuppressed Patients with Inflammatory Bowel Disease (VIP): A Multicentre, Prospective, Case-Control Study. Lancet Gastroenterol. Hepatol..

[92] Spiera E., Ganjian D.Y., Zhang X., Brenner E.J., Agrawal M., Colombel J.-F., Kappelman M.D., Kornbluth A., Ungaro R.C. (2022). Outcomes of COVID-19 Infections in Vaccinated Patients with Inflammatory Bowel Disease: Data from an International Registry. Inflamm. Bowel Dis..

[93] Schiff A.E., Wang X., Patel N.J., Kawano Y., Hanberg J.L., Kowalski E.N., Cook C.E., Vanni K.M., Qian G., Bade K.J. (2024). Immunomodulators and Risk for Breakthrough COVID-19 after Third SARS-CoV-2 mRNA Vaccine among Patients with Rheumatoid Arthritis: A Cohort Study. Ann. Rheum. Dis..

[94] Waljee A.K., Wiitala W.L., Govani S., Stidham R., Saini S., Hou J., Feagins L.A., Khan N., Good C.B., Vijan S. (2016). Corticosteroid Use and Complications in a US Inflammatory Bowel Disease Cohort. PLoS ONE.

[95] Schreiber S., Nikolaus S., Hampe J. (1998). Activation of Nuclear Factor κB in Inflammatory Bowel Disease. Gut.

[96] Brenner E.J., Ungaro R.C., Gearry R.B., Kaplan G.G., Kissous-Hunt M., Lewis J.D., Ng S.C., Rahier J.-F., Reinisch W., Ruemmele F.M. (2020). Corticosteroids, But Not TNF Antagonists, Are Associated with Adverse COVID-19 Outcomes in Patients with Inflammatory Bowel Diseases: Results from an International Registry. Gastroenterology.

[97] Alrashed F., Battat R., Abdullah I., Charabaty A., Shehab M. (2021). Impact of Medical Therapies for Inflammatory Bowel Disease on the Severity of COVID-19: A Systematic Review and Meta-Analysis. BMJ Open Gastroenterol..

[98] Sewell G.W., Kaser A. (2022). Interleukin-23 in the Pathogenesis of Inflammatory Bowel Disease and Implications for Therapeutic Intervention. J. Crohns Colitis.

[99] Ghosh S., Feagan B.G., Ott E., Gasink C., Godwin B., Marano C., Miao Y., Ma T., Loftus E.V., Sandborn W.J. (2024). Safety of Ustekinumab in Inflammatory Bowel Disease: Pooled Safety Analysis Through 5 Years in Crohn’s Disease and 4 Years in Ulcerative Colitis. J. Crohns Colitis.

[100] Habtezion A., Nguyen L.P., Hadeiba H., Butcher E.C. (2016). Leukocyte Trafficking to the Small Intestine and Colon. Gastroenterology.

[101] Zeissig S., Rosati E., Dowds C.M., Aden K., Bethge J., Schulte B., Pan W.H., Mishra N., Zuhayra M., Marx M. (2019). Vedolizumab Is Associated with Changes in Innate Rather than Adaptive Immunity in Patients with Inflammatory Bowel Disease. Gut.

[102] Edelman-Klapper H., Zittan E., Bar-Gil Shitrit A., Rabinowitz K.M., Goren I., Avni-Biron I., Ollech J.E., Lichtenstein L., Banai-Eran H., Yanai H. (2021). Lower Serologic Response to COVID-19 mRNA Vaccine in Patients with Inflammatory Bowel Diseases Treated with Anti-TNFα. Gastroenterology.

[103] Nakanishi Y., Sato T., Ohteki T. (2015). Commensal Gram-Positive Bacteria Initiates Colitis by Inducing Monocyte/Macrophage Mobilization. Mucosal Immunol..

[104] Ninnemann J., Winsauer C., Bondareva M., Kühl A.A., Lozza L., Durek P., Lissner D., Siegmund B., Kaufmann S.H.E., Mashreghi M.-F. (2022). TNF Hampers Intestinal Tissue Repair in Colitis by Restricting IL-22 Bioavailability. Mucosal Immunol..

[105] Globig A.-M., Mayer L.S., Heeg M., Andrieux G., Ku M., Otto-Mora P., Hipp A.V., Zoldan K., Pattekar A., Rana N. (2022). Exhaustion of CD39-Expressing CD8+ T Cells in Crohn’s Disease Is Linked to Clinical Outcome. Gastroenterology.

[106] Cope A.P., Londei M., Chu N.R., Cohen S.B., Elliott M.J., Brennan F.M., Maini R.N., Feldmann M. (1994). Chronic Exposure to Tumor Necrosis Factor (TNF) in Vitro Impairs the Activation of T Cells through the T Cell Receptor/CD3 Complex; Reversal in Vivo by Anti-TNF Antibodies in Patients with Rheumatoid Arthritis. J. Clin. Investig..

[107] Bosè F., Raeli L., Garutti C., Frigerio E., Cozzi A., Crimi M., Caprioli F., Scavelli R., Altomare G., Geginat J. (2011). Dual Role of Anti-TNF Therapy: Enhancement of TCR-Mediated T Cell Activation in Peripheral Blood and Inhibition of Inflammation in Target Tissues. Clin. Immunol. Orlando Fla.

[108] Buhre J.S., Pongracz T., Geisen U.M., Schubert M., Wang W., Nouta J., Obara M., Lehrian S., Rahmöller J., Petry J. (2024). Anti-TNF Therapy Impairs Both Short- and Long-Term IgG Responses after Repeated Vaccination. Allergy.

[109] Coldewey S.M., Neu C., Bloos F., Baumbach P., Schumacher U., Bauer M., Reuken P., Stallmach A. (2022). Infliximab in the Treatment of Patients with Severe COVID-19 (INFLIXCOVID): Protocol for a Randomised, Controlled, Multicentre, Open-Label Phase II Clinical Study. Trials.

[110] Bezzio C., Saibeni S., Variola A., Allocca M., Massari A., Gerardi V., Casini V., Ricci C., Zingone F., Amato A. (2020). Outcomes of COVID-19 in 79 Patients with IBD in Italy: An IG-IBD Study. Gut.

[111] Ben-Tov A., Banon T., Chodick G., Kariv R., Assa A., Gazit S. (2021). BNT162b2 Messenger RNA COVID-19 Vaccine Effectiveness in Patients with Inflammatory Bowel Disease: Preliminary Real-World Data During Mass Vaccination Campaign. Gastroenterology.

[112] Hadi Y., Dulai P.S., Kupec J., Mohy-Ud-Din N., Jairath V., Farraye F.A., Kochhar G.S. (2022). Incidence, Outcomes, and Impact of COVID-19 on Inflammatory Bowel Disease: Propensity Matched Research Network Analysis. Aliment. Pharmacol. Ther..

[113] Lev-Tzion R., Focht G., Lujan R., Mendelovici A., Friss C., Greenfeld S., Kariv R., Ben-Tov A., Matz E., Nevo D. (2022). COVID-19 Vaccine Is Effective in Inflammatory Bowel Disease Patients and Is Not Associated with Disease Exacerbation. Clin. Gastroenterol. Hepatol..

[114] Lee J.J.Y., Bernatsky S., Benchimol E.I., Kuenzig M.E., Kwong J.C., Li Q., Widdifield J. (2024). COVID-19 Vaccination Safety and Associated Health Care Utilization among Adults with Inflammatory Bowel Disease—A Population-Based Self-Controlled Case Series Analysis. BMC Gastroenterol..

[115] Brenner E.J., Weaver K.N., Zhang X., Kastl A.J., Strople J.A., Adler J., Dubinsky M.C., Bousvaros A., Watkins R., Dai X. (2024). Long-Term Effectiveness and Durability of COVID-19 Vaccination Among Patients with Inflammatory Bowel Disease. Clin. Gastroenterol. Hepatol..

[116] Elmahdi R., Ward D., Ernst M.T., Poulsen G., Hallas J., Pottegård A., Jess T. (2024). Impact of Immunosuppressive Therapy on SARS-CoV-2 mRNA Vaccine Effectiveness in Patients with Immune-Mediated Inflammatory Diseases: A Danish Nationwide Cohort Study. BMJ Open.

[117] Kennedy N.A., Janjua M., Chanchlani N., Lin S., Bewshea C., Nice R., McDonald T.J., Auckland C., Harries L.W., Davies M. (2023). Vaccine Escape, Increased Breakthrough and Reinfection in Infliximab-Treated Patients with IBD during the Omicron Wave of the SARS-CoV-2 Pandemic. Gut.

[118] Gao Y., Cai C., Wullimann D., Niessl J., Rivera-Ballesteros O., Chen P., Lange J., Cuapio A., Blennow O., Hansson L. (2022). Immunodeficiency Syndromes Differentially Impact the Functional Profile of SARS-CoV-2-Specific T Cells Elicited by mRNA Vaccination. Immunity.

[119] Veldhoen M., Bertoletti A. (2023). SARS-CoV-2 Clearance after Breakthrough Infection Correlates with Fit and Happy T Cells. Immunol. Cell Biol..

[120] Eser T.M., Baranov O., Huth M., Ahmed M.I.M., Deák F., Held K., Lin L., Pekayvaz K., Leunig A., Nicolai L. (2023). Nucleocapsid-Specific T Cell Responses Associate with Control of SARS-CoV-2 in the Upper Airways before Seroconversion. Nat. Commun..

[121] Petrone L., Sette A., de Vries R.D., Goletti D. (2023). The Importance of Measuring SARS-CoV-2-Specific T-Cell Responses in an Ongoing Pandemic. Pathogens.

[122] Moreira-Teixeira L., Redford P.S., Stavropoulos E., Ghilardi N., Maynard C.L., Weaver C.T., Freitas do Rosário A.P., Wu X., Langhorne J., O’Garra A. (2017). T Cell–Derived IL-10 Impairs Host Resistance to Mycobacterium Tuberculosis Infection. J. Immunol. Author Choice.

